# Biomechanical stress in the context of competitive sports training triggers enthesitis

**DOI:** 10.1186/s13075-021-02530-x

**Published:** 2021-06-21

**Authors:** David Simon, Arnd Kleyer, Sara Bayat, Johannes Knitza, Larissa Valor-Mendez, Marina Schweiger, Georg Schett, Koray Tascilar, Axel J. Hueber

**Affiliations:** 1 Department of Internal Medicine 3 - Rheumatology and Immunology, Friedrich-Alexander University (FAU) Erlangen-Nürnberg and Universitätsklinikum Erlangen, 91054 Erlangen, Germany; 2 Deutsches Zentrum Immuntherapie, Friedrich-Alexander University (FAU) Erlangen-Nürnberg and Universitätsklinikum Erlangen, 91054 Erlangen, Germany; 3 Rheumazentrum Erlangen, 91054 Erlangen, Germany; 4 Section Rheumatology, Sozialstiftung Bamberg, 96049 Bamberg, Germany

**Keywords:** Enthesitis, Biomechanical stress, Inflammation

## Abstract

**Objective:**

To evaluate the influence of mechanical stress on the development of immediate enthesitis.

**Methods:**

The BEAT study is an interventional study that assessed entheses in competitive badminton players before and immediately after a 60-min intensive training session. Power Doppler (PD) signal and Gray scale (GS) changes were assessed in the insertion sites of both Achilles tendon, patellar tendons, and lateral humeral epicondyles and quantified using a validated scoring system.

**Results:**

Thirty-two badminton players were included. One hundred ninety-two entheseal sites were examined twice. The respective empirical total scores for PD examination were 0.1 (0.3) before and 0.5 (0.9) after training. Mean total GS scores were 2.9 (2.5) and 3.1 (2.5) before and after training, respectively. The mean total PD score difference of 0.4 between pre- and post-training was significant (*p* = 0.0014), whereas no significant difference for the mean total GS score was observed. Overall, seven participants (22%) showed an increased empirical total PD score. A mixed effects model showed a significant increase of PD scores after training, with a mean increase per site of 0.06 (95% CI 0.01 to 0.12, *p* = 0.017).

**Conclusions:**

Mechanical stress leads to rapid inflammatory responses in the entheseal structures of humans. These data support the concept of mechanoinflammation in diseases associated with enthesitis.

## Introduction

Entheses link bones with tendons and ligaments [1]. They constitute essential components for transducing mechanical forces and providing musculoskeletal stability. It is commonly considered that mechanical overloading of entheses triggers a local inflammatory response that leads to pain and impaired locomotor function (“enthesitis”) [2]. Thus, while entheses are anatomically designed to cope with certain mechanical stress, physical challenges that surpass certain thresholds may lead to an inflammatory response and, if long-lasting, accrual of damage. Certain factors such as age or obesity may lower the individual threshold for mechanically induced inflammatory responses [3]. In addition, spondyloarthritis (SpA) including psoriatic arthritis (PsA) is characterized by enthesitis, which often is long-lasting and recurrent [4, 5].

Based on the role of enthesitis in human diseases, mechanistic studies that help to understand the concept of mechanoinflammation in humans are required. The concept of mechanically induced enthesitis is well established in disease models [6] but lags behind in humans. The concept of mechanoinflammation in humans is mostly based on anectodal observations on the tennis or golfer’s elbow or Achilles tendon pain in runners. Furthermore, the mere presence of pain at an entheseal site is not an unmistakable sign for enthesitis. Thus, it is by no means clear whether tenderness always represents inflammation. Examination of entheses by power Doppler ultrasound (PDUS), however, allows the detection of entheseal inflammation by measuring the PD signal (PD) or detecting enthesitis indirectly by the assessment of structural changes such as bony proliferations, erosions, or calcification [7].

Since the effect of mechanical stress on healthy entheses has not been examined in a systematic way, we set up a study, in which we investigated entheseal inflammatory responses to high-level and well-defined load of mechanical stress by PDUS. In this so-called BEAT study (*B*adminton *E*nthesitis *A*rthrosonography), we took advantage of competitive badminton, which exerts an overall high-level mechanical stress on the entheses and is characterized by side differences of the load to the same entheseal sites, e.g., striking hand vs. non-striking hand.

## Materials and methods

### Participants

In the interventional BEAT study, competitive players from two different badminton clubs in Germany were recruited. Only active, adult badminton players were considered for participation. Players who had psoriasis, a family history of psoriasis, chronic inflammatory low back pain, a diagnosis of a rheumatic disease, or pregnancy were not allowed to participate. All subjects included underwent a physical examination by a rheumatologist, who was blinded to the ultrasound results and performed clinical examination of 29 entheses just before and after intensive training. The training was a standardized unit of a 15-min warm-up followed by a 60-min preparation of competitive badminton (high impact training with batting, bending, and jumping). The study was approved by the ethics committee (226_18 B); all subjects provided informed consent.

### Ultrasound assessment

Immediately after each physical assessment, a PDUS examination of entheseal sites was performed. Due to the special requirements of the stop-and-go sport of badminton, we focused on entheseal sites that are subject to particularly high stress: Achilles tendon insertion, patellar tendon at the tibial insertion, and lateral humeral epicondyle (all both sides). The entheses of the Achilles tendons were evaluated in neutral zero position, whereby the player was positioned in a prone position with feet overhanging the examination table; the entheses of the patellar tendon at the tibial insertion were assessed in supine position with a full stretched leg; and the entheses of the lateral humeral epicondyle were assessed in a flexed position. All PDUS examinations were performed with a Samsung HS50 ultrasound device by an experienced investigator (AK) in accordance with the OMERACT definitions for ultrasonographic pathologies [7]. The system was equipped with a linear probe LA3-16A of the frequency range 3–16 MHz and with a linear probe LA3-14AD of the frequency range 3–14 MHz. A penetration depth of 2 cm was set. In the 2D Gray scale image, a frequency of 6.7 MHz or 4.8 MHz with a gain of 66 and a dynamic range of 96 was used. For power Doppler assessment, a frequency of 7.0 MHz and a pulse repetition frequency of 0.5 kHz were used.

### Ultrasound evaluation

All images were scored by two blinded and independent, PDUS experienced readers using a modified OMERACT score [8] on pre-recorded images in a random order. Using Gray scale (GS), the images were scored according to the severity of structural changes: (0) no abnormalities, (1) hypoechogenicity, (2) thickening and hypoechogenicity plus calcifications/enthesophytes, and (3) thickening and hypoechogenicity plus calcifications/enthesophytes and erosions. PD was used for assessing inflammation using the following scoring adapted system: (0) no PD signal, (1) one or two Doppler spots at cortical insertion, (2) more than two Doppler spots at cortical insertion and up to 2 mm from the cortical bone, and (3) extensive Doppler signal at cortical insertion. These were added to get empirical total scores for GS and PD.

### Statistical analysis

Participant characteristics were described using means and standard deviations for continuous variables, or counts and percentages for categorical variables. We expected that entheseal changes after training would be detected primarily by PD, whereas GS changes would likely be small and haphazard if any. For this reason, we did not calculate an overall total score and evaluated the GS and PD scores separately.

Pre- and post-training scores were compared using linear mixed effects models. In these models, we used the raw site scores as the dependent variable, timepoint as the fixed effect of interest, patient ID, and sites of measurement within patients as nested random effects. In further analyses, we included the site of measurement as a fixed effect, in order to assess the overall entheseal change burden by entheseal site, and also time-site interaction terms were used to assess whether a particular entheseal site was differentially affected by training.

Although mixed effects linear regression is relatively robust to violation of distributional assumptions and missing observations, due to the small size and overall sparsity of the observed changes, we also compared the pre-post training difference of enthesitis scores using permutation tests as a sensitivity analysis. For these tests, the scores were randomly shuffled to generate multiple permuted datasets while the order of the timepoint indicator was kept unchanged. This provides a sampling space of pre-post mean differences. However, using every possible permutation of the data would result in approximately 2.6 × 10^35^ datasets and was not tractable. We therefore used a Monte Carlo simulation with 10,000 replications to generate random sampling spaces consisting of randomly permuted datasets, from which an exact probability of observing a range of given pre-post mean differences can be estimated assuming the null hypothesis of equal means regardless of distributional assumptions.

All p values were two-sided and considered significant if less than 0.05 without multiplicity adjustment. The analyses were conducted using R version 4.0.1 (R Foundation for Statistical Computing, Vienna, Austria) and RStudio version 1.2.13 (RStudio Inc. Boston, MA) using the lme4 and perm packages.

## Results

### Study population

Thirty-four subjects were screened for study inclusion; two had to be excluded because of a history of psoriasis. Thirty-two badminton players (22 men, 10 women, age 31.1 ± 13.0 years) finally participated in the study. On average, they had been playing badminton for 16.2 ± 10.1 years (Table 1). Almost one in two athletes reported a history of sports injuries (15/32 (47%)) and musculoskeletal pain (13/32; 41%), especially in the lower extremity. Knee pain occurred in 11/32 players (34%), followed by Achilles tendon pain 2/32 (6%) and elbow pain 1/32 (3%). These symptoms were reported to be very mild, the mean total pain score was 1.6 ± 2.0 on a visual analogue scale (0–10), and no players were taking nonsteroidal anti-inflammatory drugs for musculoskeletal problems.

**Table 1:** Baseline characteristics

**Demographic characteristics**	
N, total	32
Females, N (%)	10 (31.3)
Age, years (mean ± SD)	31.1 ± 13.0
Height, cm (mean value ± SD)	178.6 ± 9.9
Body weight, kg (mean value ± SD)	74.7 ± 13.5
Smoking, N (%)	11 (34.4)
Alcohol, N (%)	24 (75.0)
**Concomitant diseases**	
Inflammatory bowel disease, N (%)	0
Psoriasis, N (%)	0
Uveitis, N (%)	0
Diabetes mellitus, N (%)	0
Hypertension, N (%)	2 (6.3)
**Sports history**	
Years badminton (mean ± SD)	16.2 ± 10.1

### Prevalence of enthesitis after training

Clinical examination revealed entheseal pain in only one participant before training, while four subjects reported entheseal pain after training. By ultrasound, 192 entheseal sites were examined twice. The respective mean total scores (SD) for PD examination were 0.1 (0.3) before and 0.5 (0.9) after training, while the respective GS scores were 2.9 (2.5) and 3.1 (2.5). Overall, seven participants (22%) showed an increased total PD score. The GS score increased in 3, decreased in 1, and remained unchanged in the remaining participants.

### Mixed effects model on training effect on enthesitis

**Fig. 1:**
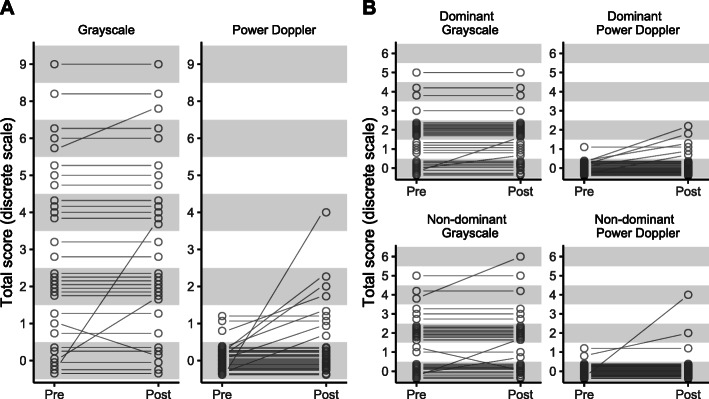
**a** Spaghetti plots depicting individual Gray scale and power Doppler ultrasound scores before and after training and **b** calculated separately for the dominant and non-dominant sides

The overall course of the individual participant scores is depicted in Fig. 1. A mixed effects model showed that the increase in PD scores after training was significant, with a mean increase in the site scores of 0.06 (95% CI 0.01 to 0.12, p = 0.017). The model for the GS scores did not show a significant difference between pre- and post-training scores (mean increase 0.03 (95% CI, −0.09 to 0.15, p = 0.645)). Assessment of the entheseal sites in this model indicates higher structural changes in the Achilles tendon insertion compared to the lateral epicondyle (Table 2).

**Table 2:** Model coefficients

Dependent	Term	Estimate (95% CI)	p
Gray scale item score	(Intercept)	0.10 (−0.08 to 0.28)	0.279
	Time post-training^a^	0.03 (−0.09 to 0.15)	0.645
	Patella^b^	0.12 (−0.04 to 0.27)	0.135
	Achilles^b^	1.04 (0.89 to 1.19)	<0.001
Power Doppler item score	(Intercept)	0.00 (−0.06 to 0.06)	>0.99
	Time-post^a^	0.06 (0.01 to 0.12)	0.017
	Patella^b^	0.02 (−0.06 to 0.09)	0.670
	Achilles^b^	0.03 (−0.04 to 0.11)	0.394

^a^Reference level: pre-training, ^b^reference level: elbow

### Sensitivity analyses of training effect on enthesitis

The sensitivity analyses using permutation tests did not refuse the overall conclusions from the mixed model analyses about pre-post change. The mean total PD score difference of 0.4 between pre- and post-training was significant with a p-value of 0.0014, whereas the p-value for the mean total GS score difference of 0.2 was 0.63.

### Distribution of enthesitis according to handedness

Finally, among the 7 participants that showed an increase in the PD score, 5 showed these changes on the dominant side, while 2 participants showed changes on the non-dominant side (Fig. 1). In addition, in all 3 participants with a post-training increase in PD activity of the elbow, changes were observed exclusively on the dominant side.

## Discussion

In this study, we show that defined mechanical stress can lead to the development of entheseal inflammation in humans. Thus, we observed a significant increase in PD activity at entheseal sites in competitive badminton players after standard training. However, this observed effect was primarily a moderate increase in some participants; in the majority of players, musculoskeletal loading had no impact. Studies on mechanoinflammation in humans have so far been focused on single sites (Achilles or patellar tendon) and related to tendinitis rather than enthesitis [9–11]. So far, no comprehensive study has been done that included multiple entheseal sites after full-body physical challenge.

Interestingly, the concept of mechanoinflammation is already well understood for other musculoskeletal abnormalities. Thus, comprehensive magnetic resonance imaging studies in athletes have already shown that mechanical stress is associated with the development of bone marrow edema [12, 13]. Several results from this study are interesting with regard to the mechanoinflammatory concept of enthesitis: (i) Signs of entheseal inflammation developed fast, as early as 1 h after high-level strain, indicating an immediate stress response to mechanical strain. Furthermore, (ii) despite similar mechanical load associated with standard training, the susceptibility to develop entheseal inflammation differed among the subjects, suggesting an individual threshold for mechanoinflammation. Finally, (iii) entheses at the dominant site were more frequently involved than their contralateral counterparts, indicating that even in one and the same individual the mechanical load matters in triggering inflammation.

From our study, we do not know how long entheseal inflammation lasted after mechanical challenge. It is likely, however, that inflammation spontaneously resolved as the badminton players represented healthy individuals. In PsA and SpA patients, such inflammatory stress response in the entheses however may be prolonged and enhanced. It is conceivable that the individual threshold for mechanically induced inflammatory responses in the entheses is genetically determined and maybe lower in PsA and SpA as compared to healthy subjects. The higher prevalence of entheseal lesions in psoriasis patients [14] and the observation that mechanical factors facilitate the translation from psoriatic skin to joint disease may support that notion [15, 16].

The results from the BEAT study contribute to a deeper understanding of enthesitis development and to a refinement of the concept of mechanoinflammation. This concept, which has evolved from preclinical findings, suggests that mechanical stress leads to release mediators such as PGE2, IL-17, or TNF-α and chemokines such as CXCL1 and CCL2 [5, 6]. The release of these molecules causes vasodilation, recruitment of neutrophils, and other innate immune cells to entheseal sites. If chronic, this process initiates differentiation of mesenchymal cells that contribute to the formation of function-limiting entheseal lesions [4]. However, data from human biopsy studies are urgently needed for the final confirmation of this concept. Fortunately, some important studies are underway that will provide important clarifying data. Of note, even in this healthy study group, structural changes were found, which suggest that repeated mechanical stress can lead to the accrual of entheseal damage.

A limitation of the study is the limited number of badminton players investigated and the lack of inclusion of a patient group susceptible for enthesitis such as psoriasis or PsA patients. On the other hand, training of such individuals would essentially differ from the homogeneous training these athletes are performing and would thus not have allowed a reasonable comparison. Also, the cross-sectional study design does not allow to assess potential adaption of the entheseal architecture to mechanical stress over time. Our statistical evaluation with a linearity assumption for the bounded discrete ultrasound scores is another shortcoming of this analysis. We attempted to overcome this shortcoming using sensitivity analyses with permutation tests and confirmed the overall conclusions from mixed models.

## Conclusions

In conclusion, the BEAT study revealed that mechanical stress induces an inflammatory response in the entheseal organ in healthy individuals in vivo. These findings support the important role of mechanical factors in the development of enthesitis, as shown previously in experimental models, and indicate that mechanoinflammation is an important factor in diseases characterized by enthesitis such as PsA.

## Data Availability

All data generated or analyzed during this study are included in this article.
